# Sub-arc collimator angle optimization based on the conformity index heatmap for VMAT planning of multiple brain metastases SRS treatments

**DOI:** 10.3389/fonc.2022.987971

**Published:** 2022-09-06

**Authors:** Jiuling Shen, Zhitao Dai, Jing Yu, Qingqing Yuan, Kailian Kang, Cheng Chen, Hui Liu, Conghua Xie, Xiaoyong Wang

**Affiliations:** ^1^ Department of Radiation and Medical Oncology, Zhongnan Hospital of Wuhan University, Wuhan, China; ^2^ Hubei Radiotherapy Quality Control Center, Wuhan University, Wuhan, China; ^3^ National Cancer Center/National Clinical Research Center for Cancer/Cancer Hospital and Shenzhen Hospital, Chinese Academy of Medical Sciences and Peking Union Medical College, Shenzhen, China

**Keywords:** collimator angle optimization, volumetric modulated arc therapy, stereotactic radiosurgery, multiple brain metastases, sub-arc

## Abstract

**Objective:**

The aim of this study was to investigate the impact of collimator angle optimization in single-isocenter coplanar volume modulated arc therapy (VMAT) stereotactic radiosurgery (SRS) for multiple metastases with respect to dosimetric quality and treatment delivery efficiency. In particular, this is achieved by a novel algorithm of sub-arc collimator angle optimization (SACAO).

**Methods:**

Twenty patients with multiple brain metastases were retrospectively included in this study. A multi-leaf collimator (MLC) conformity index (MCI) that is defined as the ratio of the area of target projection in the beam’s eye view (BEV) to the related area fitted by MLC was applied. Accordingly, for each control point, 180 MCI values were calculated with a collimator angle interval of 1°. A two-dimensional heatmap of MCI as a function of control point and collimator angle for each full arc was generated. The optimal segmentation of sub-arcs was achieved by avoiding the worst MCI at each control point. Then, the optimal collimator angle for each sub-arc would be determined by maximizing the summation of MCI. Each patient was scheduled to undergo single-center coplanar VMAT SRS based on either the novel SACAO algorithm or the conventional VMAT with static collimator angle (ST-VMAT). The dosimetric parameters, field sizes, and the monitoring units (Mus) were evaluated.

**Results:**

The mean dose-volumetric parameters for the target volume of SACAO were comparable to ST-VMAT, while the conformity index (CI), homogeneity index (HI), and gradient index (GI) were reduced by SACAO. Improved sparing of organs at risk (OARs) was also obtained by SACAO. In particular, the SACAO method significantly (*p* < 0.01) reduced the field size (76.59 ± 32.55 vs. 131.95 ± 56.71 cm^2^) and MUs (655.35 ± 71.99 vs. 729.85 ± 73.52) by 41.11%.

**Conclusions:**

The SACAO method could be superior in improving the CI, HI, and GI of the targets as well as normal tissue sparing for multiple brain metastases SRS. In particular, SACAO has the potential of increasing treatment efficiency in terms of field size and MU.

## 1 Introduction

**Figure 1:**
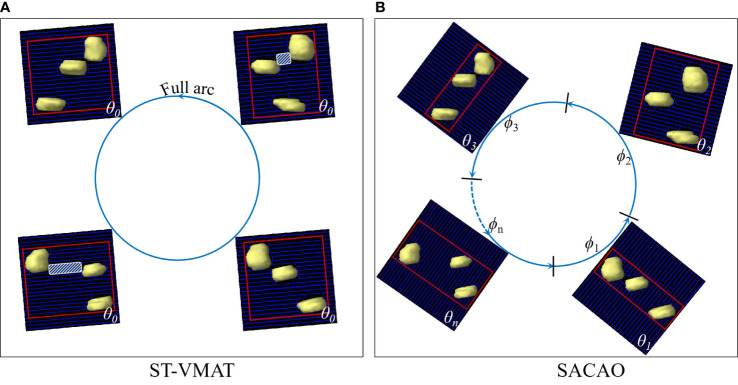
BEV of target volumes with multiple targets fitted by MLCs with ST-VMAT **(A)** and SACAO **(B)**. The red rectangles depict field size in both figures. The white shaded areas display the island blocking problem when two or more targets share the same MLCleaf pair.

Volume-modulated arc therapy (VMAT) has been widely used in clinical practice due to its better dose distribution and higher treatment efficiency (1–6). VMAT technology simultaneously combines many degrees of freedom, such as beam energy, couch rotation, gantry rotation speed, multileaf collimator (MLC) motion, and dose rate modulation (1, 2). Among the aforementioned degrees of freedom, collimator rotation has been shown to have an important impact on the quality of the VMAT planning (7–10), since it enables the optimization of MLC to shape a conformal dose distribution for the target volume and block normal organs properly. However, in the current VMAT implementation, the collimator angle is kept static in each arc, which we term standard VMAT (ST-VMAT) for clarity (as shown in **Figure 1A**).

The optimal choice of the collimator angle has been controversial for the VMAT planning. Treutwein (11) and Otto et al. (1) have demonstrated that a collimator angle of 45° has been found to be suitable for prostate cancer in most cases. Ward et al. (12) and Ahnd et al. (13) showed that the VMAT optimization involving sectional optimization of collimator angle could provide delivery efficiency and dosimetric improvements. Furthermore, dynamic collimator angle optimization based on target anatomy has been widely investigated by VMAT with different goals and approaches, and presented the potential to improve dose distribution and/or delivery efficiency. Zhang et al. (14) provided a collimator trajectory optimization method for the treatment of paraspinal lesions based on principal component analysis (PCA) calculated from the beam’s eye view (BEV) to the spinal cord. The method allows for collimator angle rotations during the beam delivery. However, the use of PCA does not necessarily result in optimal angles. Bokrantz et al. (15) provide an optimization method that transfers the collimator trajectory per fluence map to a trajectory per control point of the segmented VMAT plan, which may be integrated in a treatment planning system. MacDonald et al. (16, 17) propose a method to minimize the exposed area of non-target anatomy in a previous study by generating automated fixed couch trajectory and dynamic collimator trajectories, respectively. Murtaza et al. (18) suggested that a better dose distribution in the pelvis could be obtained by dynamically rotating the collimator *via* allowing the MLC to track the trajectory of the prostate in the course of the treatments.

In patients with multiple brain metastases, a paradigm of isometric radiotherapy would facilitate reproducibility of treatment and accurate dose assessment of targets and organs at risk. However, when treating multiple targets or targets with irregular shapes that include concavities, the projection of the target(s) as seen from the BEV will change as a function of control point. In particular, when the tumors are aligned along the motional direction of MLC, the normal tissues between the lesions may not be well shielded, known as the “island blocking problem” (9, 19). Island blocking, also referred to as a high-dose bridge, may result in an area of healthy tissue that could not be shielded. The optimization of the collimator angle is a critical approach to improving this problem (8–10, 12, 17). In order to protect the patient’s memory and cognitive function as much as possible, we proposed a novel algorithm of sub-arc collimator angle optimization (SACAO), in which the segmentation of sub-arcs and the optimal collimator angles were determined based on patient-specific target in the BEV view (as shown in **Figure 1B**). The performance of this technique was tested, and its dosimetric quality and treatment delivery efficiency were compared to the conventional VMAT (ST-VMAT) plans.

## 2 Materials and methods

### 2.1 Patient selection

This retrospective study was approved by the institutional review board. A total of 20 patients with two to five brain metastatic lesions previously treated at the radiotherapy center of Zhongnan Hospital of Wuhan University from October 2019 to September 2020 were included in this study. The patient characteristics are detailed in **Table 1**. The prescription dose of all the patients was 30 Gy in five fractions. The gross tumor volume (GTV) was defined by experienced radiation oncologists on planning CT images fused with MRI. A 0.3-cm margin was added to create the planning target volume (PTV) to account for setup uncertainties or motion through the fractionated treatment. The median PTV was 19.6 cc (range, 8.2–48.2 cc). The distance between lesions was defined as the minimum range of their boundaries at each of the three orthogonal directions. The medians of maximum distance between each lesion (*R*_*max*_ ) in the left–right (*X*), anterior–posterior (*Y*), and inferior–superior (*Z*) directions were 8.8 cm (range, 3.0–12.5 cm), 7.5 cm (range, 3.8–10.8 cm), and 10.2 cm (range, 4.3–17.6 cm), respectively.

**Table 1:** Patient characteristics.

Patient ID	Number of lesions	Volume of each lesion (cc)	Total volumes (cc)	*R*_max_ (cm)		
				*X*	*Y*	*Z*
1	2	10, 9.3	19/3	7.8	8.8	10.0
2	2	6.4, 11.4	17.8	4.5	5.8	11.6
3	2	11.3, 7.9	19.2	8.3	7.0	12.5
4	4	16.8, 3.7, 4.8, 15	40.3	9.1	10.8	17.6
5	2	7.8, 18	25.8	3.0	8.1	11.0
6	3	9.9, 6.7, 3	19.6	8.5	7.3	5.7
7	2	2.8, 5.4	8.2	12.5	6.4	15.0
8	2	10, 5.4	15.4	7.0	8.2	10.7
9	2	7.7, 10.3	18	3.2	7.0	4.3
10	3	13.7, 10.5, 7	31.2	11.9	7.4	5.3
11	2	13.4, 8.2	21.6	3.8	3.9	8.8
12	5	4.6, 8.8, 6.9, 2.2, 6.4	28.9	10.7	7.8	10.0
13	4	14.4, 9.8, 13.1, 7.3	44.6	12.0	7.8	11.5
14	2	4.3, 13.2	17.5	6.0	7.5	6.9
15	2	14.8, 8.3	23.1	4.2	3.8	9.9
16	3	10.6, 8.7, 12	31.3	9.2	5.0	13.6
17	2	7.2, 12.4	19.6	11.2	10.0	13.0
18	3	5.9, 5.8, 5.5	17.2	10.8	7.3	10.0
19	3	6.4, 8.6, 3.6	18.6	10.4	9.3	10.3
20	4	15.8, 8.1, 15.2, 9.1	48.2	10.6	9.7	8.3

### 2.2 The SACAO method

**Figure 2:**
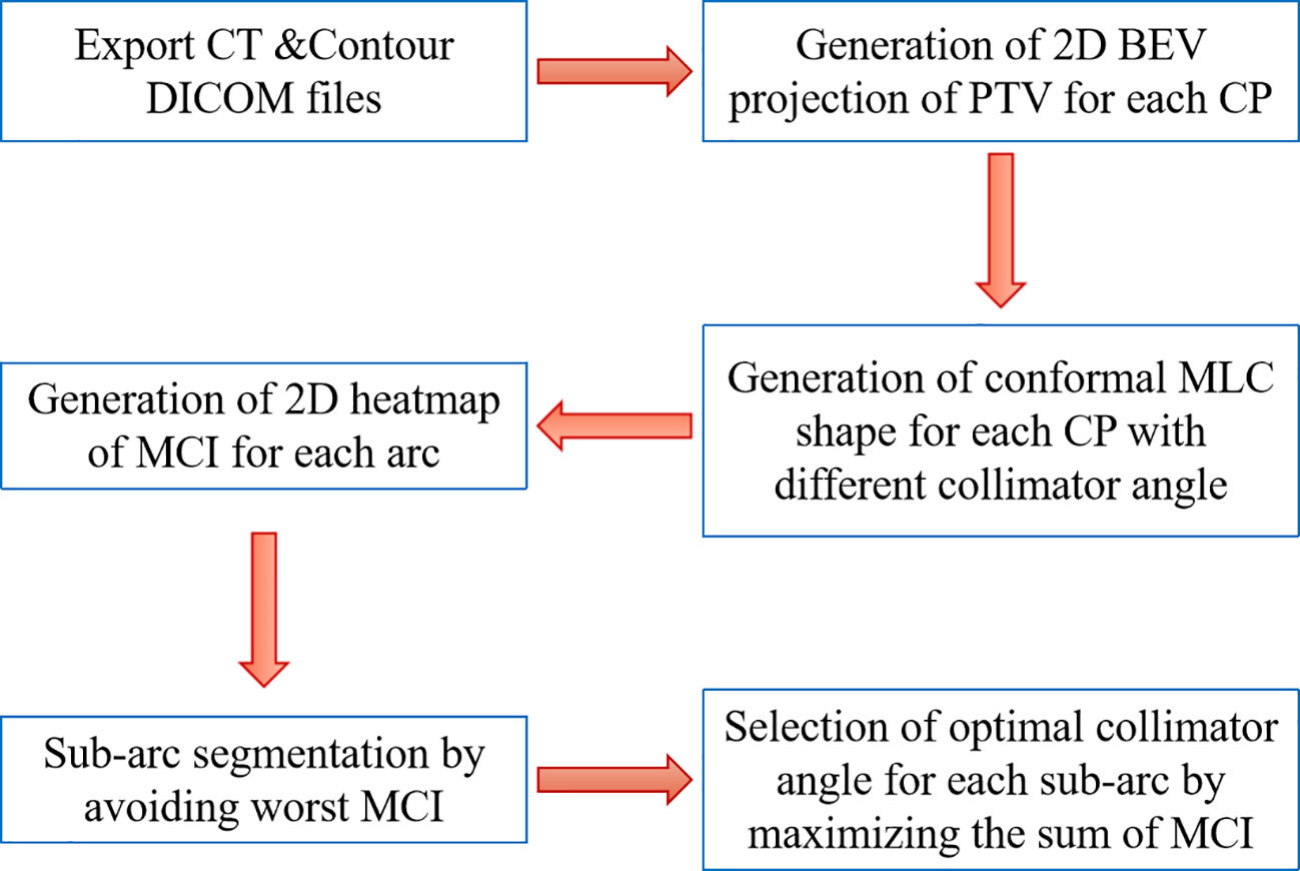
Flowchart of sub-arc collimator angle optimization (SACAO).


**Figure 3:**
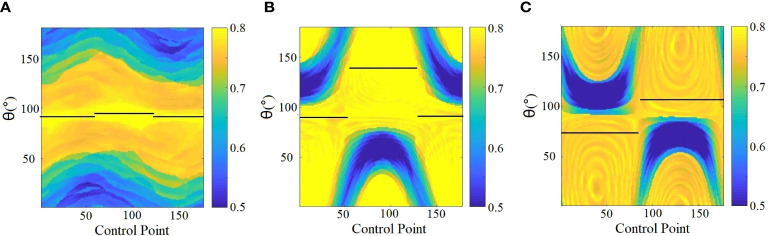
Determination of sub-arc segmentation and related collimator angles (depicted with black solid lines) based on the SACAO method for three patients **(A–C)**. The color scale represent MCI values, and the black solid line indicated optimal collimator angle for each sub-arc.

A flowchart of the proposed SACAO algorithm is shown in **Figure 2**. The SACAO algorithm was coded using MATLAB (R2017b, Mathworks, Inc., Natick, MA), and there were six main methodological processes involved:

- The CT and Contour DICOM files were exported from treatment planning system (TPS) and imported to the SACAO program.
- Two-dimensional BEV projections of the PTV for each control points at the iso-plane was calculated with spacing of 2°/CP.
- Generating conformal MLC shape for each CP with collimator angle ranging from 0° to 180°.
- Generating a 2D heatmap of MCI for each arc: an MLC conformity index (MCI) was used to quantify the sparing of normal tissue. It is defined as:

(1) $$MCI=\frac{A_{TP}}{A_{MLC}}$$

where *A*_*TP*_ is the 2D BEV projection of PTV for each control point (CP) and *A*_*MLC*_ is the area shaped by MLC. *MCI* was calculated separately for each CP with collimator angle ranging from 0° to 180°; thus, the 2D heatmap of MCI for each arc was produced (as shown in **Figure 3**).

e. Sub-arc segmentation by avoiding worst MCI: the final sub-arc could be determined by avoiding the worst MCI on the 2D heatmap, requiring consecutive gantry angles of more than 30° and less than 10 sub-arcs (**Figure 3**).
f. The sum of MCI for each sub-arc with a different collimator angle ranging from 0° to 180°; the optimal collimator for each sub-arc with the highest sum of MCI was selected.

### 2.3 Treatment planning

Both ST-VMAT and SACAO plans with two full arcs were created for each case in the Eclipse™ 13.5 (Varian Medical Systems, Palo Alto, CA) TPS. Utilizing 6 MV flattening filter free (FFF) photon beams from Varian Trilogy™ LINAC with Millennium™ 120 MLC (Varian Medical Systems, Palo Alto, CA), dose optimization and calculations were done in Eclipse TPS for all of the plans. The isocenter was located at the geometric center of the PTV. Each plan resulted from dual-arc with a gantry and rotation of 179°–181°–179°. The ST-VMAT plans that were optimized with a collimator angle of 5° and 355° for the two arcs (as shown in **Figure 1A**) were those used in the actual treatment of the patients. The algorithms of dose-volume optimizer and progressive resolution optimizer were used for dose optimizations, and the anisotropic analytical algorithm was adopted for final dose calculations. For each patient, the SACAO plan was optimized with identical dosimetric constraints but with a different collimator angle for each sub-arc (as shown in **Figure 1B**). Both ST-VMAT and SACAO plans were normalized so that 95% of the target volume was covered by prescription dose.

### 2.4 Plan comparison

The dose-volume histogram (DVH) data of the PTVs and the organs at risk were analyzed. The coverage and the minimum dose (*D*_*min*_ ), maximum dose (*D*_*max*_ ), and mean dose (*D*_*mean*_ ) of PTV were categorized for plan evaluation. Moreover, some specific dose-volume metrics such as *V*_100*%*_ , *V*_50*%*_ , *D*_2*%*_ ,  *D*_50*%*_ ,  and *D*_98*%*_ were also evaluated. Meanwhile, the conformity index (CI), homogeneity index (HI), and gradient index (GI) were also used to quantify the plan quality. CI, HI, and GI (20) were calculated as follows:


(2) $$CI=\frac{V_{ref}}{V_{T}}$$

(3) $$HI=\frac{D_{2}-D_{98}}{D_{p}}$$

(4) $$GI=\frac{V_{50\%}}{V_{100\%}}$$

where *V*_*ref*_ represents the volume covered by prescription isodose line (PIDL), *V*_*T*_ represents the target volume, *D*_*x%*_ is the dose that covers *x%* volume of PTV, *D*_*p*_ is the prescription dose of target, and *V*_*x%*_ is the absolute volume of tissue covered by *x%* of PIDL. Values of CI closer to 1.0 indicate greater conformity and values of HI close to zero indicate better homogeneity. For the SBRT plan, a smaller value of GI means steeper dose fall-off and better normal tissue sparing.

For the normal tissues, the *D*_*max*_ , *D*_*mean*_ , and organ-specific dosimetric parameters were analyzed. The volume of normal brain (brain minus PTV) covered by 5, 10, 12, and 30 Gy isodose lines (*V*_5_ , *V*_10_ , *V*_12_ , and *V*_30_ ) was compared. Additionally, the total monitor units (MUs) and the averaged field sizes (FS) for the two plans were compared. FS was calculated as follows:


(5) $$FS=\sum_{i=0}^{n}\frac{\phi_{i}}{\Phi }\cdot fs_{i}$$

For the SACAO plan, *ϕ*_*i*_ and *fs*_*i*_ are the gantry angle range and field size for each sub-arc, respectively. Φ=358^∘^ for one full arc with a gantry rotation of 179°–181° or 181°–179°.

### 2.5 Statistical analysis

Statistical analysis was performed using SPSS 22.0 (SPSS Inc., Chicago, IL). Two-sided paired *t*-tests and Mann–Whitney *U* tests were used to analyze differences between the two algorithms. *P*-value less than 0.05 was considered statistically significant.

## 3 Results

### 3.1 Sub-arc segmentation and collimator angle optimization

Twenty patients with two to five brain metastatic lesions were studied in this work. The optimized sub-arc segmentations and related collimator angles of each sub-arc were obtained from the SACAO algorithm based on the 2D heatmap of MCI for each arc. For instance, **Figure 3** demonstrated the heatmap of MCI as a function of gantry (control point) and collimator angles for three clinical cases with two lesions. The color scale represented MCI values, and the MCI value ranged from 0.5 to 0.8. The optimal collimator angle for each sub-arc was depicted with a black solid line. After optimization by the SACAO algorithm, the average number of sub-arcs in one full arc with SACAO was 3.25, ranging from 2 to 5, and the details are listed in **Table 2**.

**Table 2:** Optimized sub-arc segmentation and related collimator angles in one full arc based on the SACAO method for 20 patients.

Patient no.	No. of sub arcs	Gantry range (°)/CA (°) for each sub-arc
1	3	179-71/91, 71-293/95, 293-181/91
2	2	179-325/87, 325-181/93
3	4	179-145/32, 145-1/142, 1-329/150, 329-181/65
4	3	179-73/90, 73-281/139, 281-181/91
5	3	179-139/30, 139-299/130, 299-181/144
6	4	179-149/90, 149-319/34, 319-275/144, 275-181/90
7	4	179-49/90, 49-349/77, 349-259/90, 259-181/105
8	4	179-57/53, 57-357/160, 357-239/123, 239-181/22
9	3	179-11/160, 11-335/175, 335-181/15
10	2	179-9/74, 9-181/107
11	2	179-351/109, 351-181/72
12	4	179-77/56, 77-291/90, 291-255/116, 255-181/106
13	3	179-71/40, 71-351/25, 351-181/20
14	2	179-280/106, 280-181/90
15	4	179-65/123, 65-5/31, 5-241/57, 241-181/158
16	4	179-149/90, 149-81/35, 81-311/52, 311-181/83
17	2	179-11/130, 11-181/10
18	3	179-119/50, 119-59/153, 59-181/53
19	4	179-29/80, 29-351/90, 351-211/20, 211-181/90
20	4	179-131/135, 131-69/0, 69-277/24, 277-181/137

### 3.2 Dosimetric index evaluation

**Figure 4:**
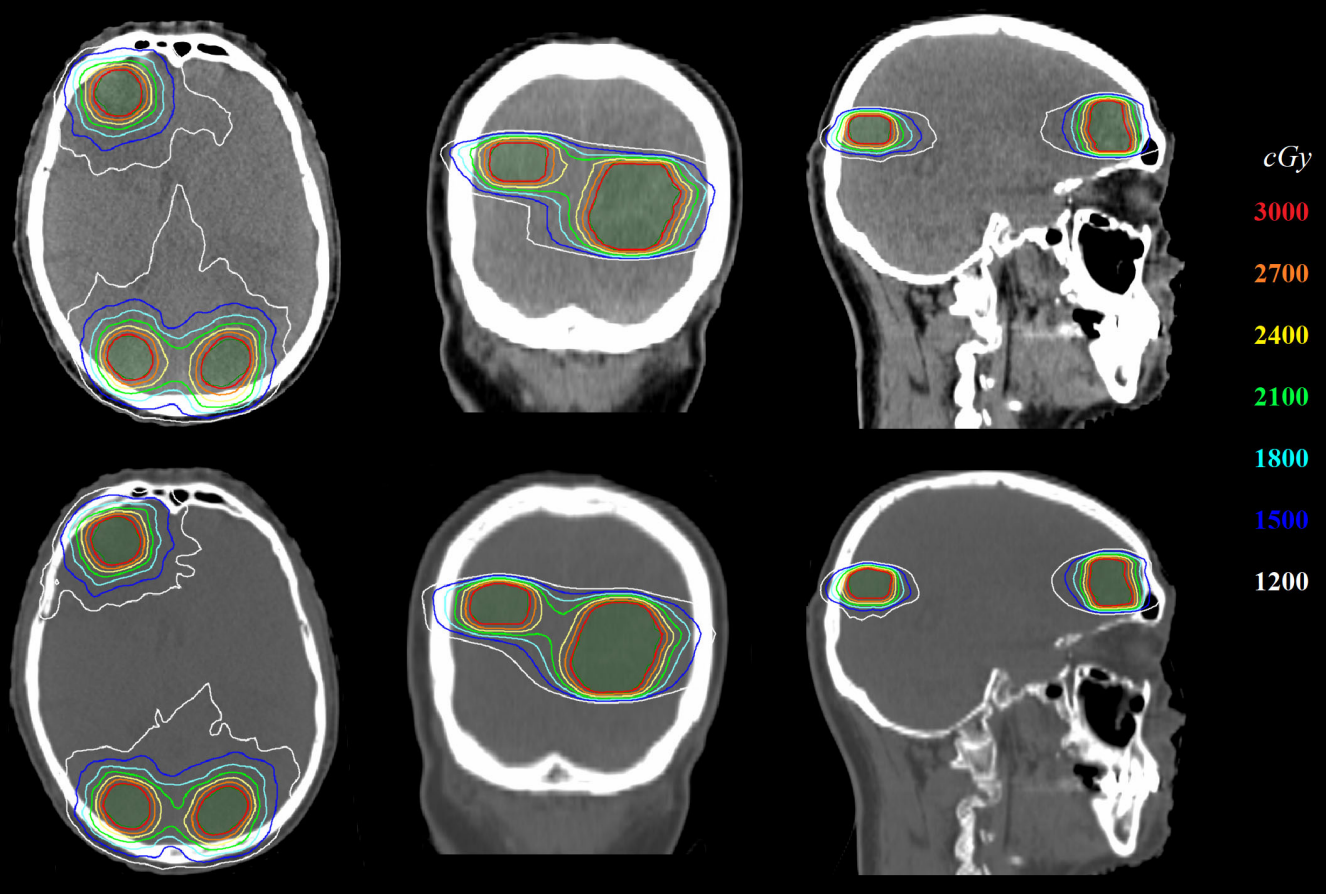
Comparison of 2D dose distribution between ST-VMAT (upper row) and SACAO (lower row) for one representative case.

**Figure 4** shows the comparison of 2D dose distributions between ST-VMAT (upper row) and SACAO (lower row) for one representative case with three lesions. Better dose conformity was achieved in the plan with SACAO. In this case, the SACAO plan improved normal brain sparing while maintaining target coverage compared to the ST-VMAT plan.

Quantitatively, the patient-averaged values of dose-volumetric parameters of ST-VMAT and SACAO for PTV and OARs in terms of mean, standard deviation (SD), and related *p* values are shown in **Tables 3**, **4**, respectively. The patient-averaged values of coverage, *D*_*min*_ , *D*_*min*_ , *D*_*min*_ , *V*_100*%*_ , *V*_50*%*_ ,  *D*_2*%*_ ,  *D*_50*%*_ ,  *D*_98*%*_, and HI were similar for the two methods, while CI and GI were improved by the SACAO method with statistical significance. In **Table 4**, it was indicated that SACAO plans presented a better sparing of OARs. For example, normal brain tissue minus PTVs showed significantly lower *D*_*max*_ and *D*_mean_ , as well as the *D*_*max*_ in the brainstem, left eye, right lens, right and left optic nerve, optic thalamus, and skin than those in ST-VMAT (*p* < 0.05 ).

**Table 3:** Dosimetric statistics of PTV for 20 patients in terms of mean, standard deviation (SD), and related *p*-values.

Metrics	ST-VMAT (*x* ± SD)	SACAO (*x* ± SD)	*p*-values
Coverage (%)	95.9 ± 0.8	96.4 ± 0.7	0.027
D*_min_* (cGy)	2,696.5 ± 102.9	2,719.8 ± 73.2	0.283
D*_max_* (cGy)	3,292.2 ± 49.4	3,236.8 ± 45.0	<0.001
D*_mean_* (cGy)	3,067.0 ± 221.8	3,097.9 ± 11.0	0.542
V*_100%_* (cc)	35.9 ± 22.7	33.8 ± 22.3	<0.001
V*_50%_* (cc)	215.2 ± 128.2	202.3 ± 133.7	0.024
D*_2%_* (cGy)	3,216.0 ± 28.5	3,173.8 ± 24.0	<0.001
D*_50%_* (cGy)	3,126.8 ± 14.3	3,104.1 ± 12.2	<0.001
D*_98%_* (cGy)	2,979.7 ± 11.1	2,976.8 ± 7.6	0.154
Homogeneity index (HI)	0.08 ± 0.01	0.06 ± 0.01	<0.001
Conformity index (CI)	1.15 ± 0.12	1.07 ± 0.06	<0.001
Gradient index (GI)	6.38 ± 1.33	5.90 ± 1.21	<0.001

*p* < 0.05 was considered statistically significant.

**Table 4:** Dosimetric statistics of OARs for 20 patients in terms of mean, standard deviation (SD), and related *p*-values.

OARs	Metrics	ST-VMAT (x ± SD)	SACAO (x ± SD)	p values
Brain-PTVs	D*_max_* (cGy)	3,248.6 ± 55.1	3,134.1 ± 229.6	0.050
	D*_mean_* (cGy)	3,248.6 ± 55.1	672.0 ± 223.8	0.001
	D*_mean_* (cGy)	697.1 ± 204.4	649.3 ± 204.5	<0.001
	V*_10Gy_* (cc)	317.1 ± 152.5	295.0 ± 162.8	0.006
	V*_12Gy_* (cc)	230.5 ± 127.1	215.4 ± 131.7	0.006
	V*_30Gy_* (cc)	4.3 ± 2.9	2.6 ± 1.9	<0.001
Brainstem	D*_max_* (cGy)	1,229.6 ± 731.7	1,169.7 ± 738.5	0.141
	D*_mean_* (cGy)	485.7 ± 391.8	482.2 ± 389.9	0.851
	D*_0.5cc_* (cGy)	965.5 ± 673.5	1,004.6 ± 674.4	0.309
Eyes L	D*_max_* (cGy)	702.0 ± 379.0	601.7 ± 363.7	0.009
Eyes R	D*_max_* (cGy)	721.8 ± 355.3	656.5 ± 360.9	0.071
Lens L	D*_max_* (cGy)	415.3 ± 246.2	353.7 ± 230.7	0.062
Lens R	D*_max_* (cGy)	373.8 ± 238.5	321.7 ± 216.4	0.034
Optical nerve L	D*_max_* (cGy)	728.5 ± 625.1	633.5 ± 623.2	0.018
	D*_mean_* (cGy)	519.5 ± 436.1	455.2 ± 394.9	0.062
	D*_0.2cc_* (cGy)	552.8 ± 438.4	482.0 ± 396.0	0.060
Optical nerve R	D*_max_* (cGy)	771.1 ± 680.0	671.4 ± 667.3	0.006
	D*_mean_* (cGy)	684.0 ± 422.8	459.2 ± 395.0	0.010
	D*_0.2cc_* (cGy)	625.3 ± 634.0	527.1 ± 619.5	0.001
Optic chiasma	D*_max_* (cGy)	1,048.0 ± 616.3	847.6 ± 488.9	0.001
	D*_mean_* (cGy)	684.0 ± 422.8	566.4 ± 342.6	0.006
	D*_0.2cc_* (cGy)	841.7 ± 500.8	674.0 ± 403.8	0.001
Skin	D*_max_* (cGy)	3,298.2 ± 46.4	3,144.5 ± 30.7	<0.001
	D*_mean_* (cGy)	3,298.2 ± 46.4	300.6 ± 118.2	<0.001
	D*_10cc_* (cGy)	3,144.5 ± 30.7	3,117.4 ± 27.8	<0.001

p < 0.05 was considered statistically significant.

### 3.3 MUs and field size

**Figure 5:**
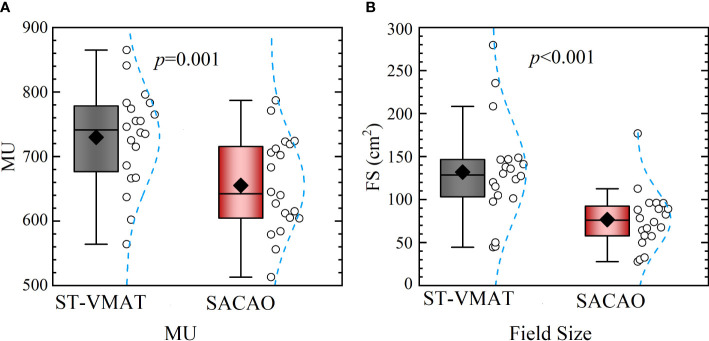
Statistical comparison of MUs **(A)** and field sizes **(B)** between ST-VMAT and SACAO plans for all patients. For SACAO plans, the field sizes are weighted average of sub-arcs for each patient.

The statistical comparison of MU and field size between ST-VMAT and SACAO plans for the 20 patients is shown in **Figure 5**. The mean field size of the SACAO plans was dramatically reduced by 41.11% (76.59 ± 32.55 vs. 131.95 ± 56.71 cmcript2, with *p*<0.01 ). In addition, the statistical reduction of MUs by SACAO plans (655.35 ± 71.99 vs. 729.85 ± 73.52 MUs, with *p*<0.001 ) was also observed. Reduction in MUs improves the plan delivery efficiency, resulting in the decreased treatment time. Moreover, less MUs, for the same prescription dose, give less out-of-field doses to the patient.

## 4 Discussion

Here, we have designed a novel SACAO algorithm for single isocentric coplanar VMAT irradiation for multiple brain metastases. In this method, the number of sub-arcs and the corresponding collimator angle were not fixed, which was patient-specific and determined by adapting the shape, size, and orientation at each lesion projection. Under the dosimetric evaluation of 20 patients with multiple brain metastases, it was found that the SACAO algorithm was able to provide better target conformity and OAR sparing than the ST-VMAT technique. At the same time, SACAO only required smaller field sizes and less MUs.

The island blocking issues, when two or more targets share the same MLC leaf pair, resulting in an area of normal brain tissue that is not blocked by MLCs, are a key parameter in IMRT and VMAT optimization for multiple brain metastases. Proper collimator angle can minimize the island blocking issue and improve plan quality. HyperArc is a technology based on a digital accelerator developed in recent years, which uses special fixed devices for immobilization. It plans to use a full arc plus several half arcs with fixed couch angles for non-coplanar delivery. At present, it has been proved that it has dosimetric advantages in the application of multiple brain metastases radiotherapy (9, 21, 22). Hyper-arc technology has its own MLC angle optimization, but in the delivery of one arc, the MLC angle is still fixed. Although it is not practical to combine the method proposed in this study with HyperArc technology due to the limitation of accelerator hardware, it deserves further study on more advanced radiotherapy unit. Ohira et al. (9) evaluated the dosimetric effects of collimator optimization in multiple brain metastases SRS with HyperArc, which indicated that the collimator-optimized plan resulted in less complexity of MLC, lower MUs, and shorter beam-on time. The collimator optimization in the HyperArc planning reduced doses to brain tissues and improved the treatment efficacy. Unfortunately, HyperArc needs special accelerators and immobilization devices, and is not available for most institutes. It is worth mentioning that our method can be applied to conventional accelerators, which is more accessible. In addition, HyperArc usually needs multiple rotation angles of the treatment couch, which puts forward higher requirements for patient immobilization and image guidance. In this study, based on the two-dimensional MCI heatmap generated according to the patient-specific anatomy, the sub-arc segmentation and the rotation angle optimization for each individual sub-arc were determined, which is less subjective. It would take full advantage of the MLC angle in a multi-target plan and diminish the “island blocking issue”. In the future, we may combine the SACAO method with HyperArc to obtain further dosimetric improvement.

Several studies have analyzed the anatomical structure information of patients and the geometric constraints of accelerators, and calculated the relevant objective functions such as the conformal index of the target area under different combinations of treatment couch, gantry, and MLC angles of VMAT. Then, a three-dimensional combined optimization of dynamic axes (CODA) function space was generated to find the optimal path of trajectory. Moreover, the dynamic couch rotation in VMAT (DCR-VMAT) technology was developed to dynamically adjust and optimize the rotation angles of treatment couch and MLC (23, 24). Furthermore, several literatures reported dividing the conventional full arc into several sub-arcs and optimizing the collimator angle within each sub arc. Of these, collimator rotation based on sub-arcs is a feasible method in clinical practice (8, 10, 13).

In comparison to the ST-VMAT plan, the SACAO algorithm allowed each pixel in the target to obtain a dose modulation involving more degrees of freedom, offering the possibility to obtain better CI and HI. Alternatively, in the SACAO algorithm, the smaller field size would reduce dose leakage and transmission, which means lower dose exposure to the organs at risk. Previous research has also attempted to use the angular conformation of the collimator for optimizing the VMAT scheme to solve the island blocking problems. Ahn et al. (13) and Kim et al. (8) uniformly divided one full arc into several sub-arcs, and collimator angles for each sub-arc were preset. It was found that a smaller field size and less MUs could be achieved by choosing the proper collimator angles for each sub-arc, which is consistent with the current work. If the segmentation of the sub-arc could be determined individually rather than *via* simple uniform division, it would further improve the quality of the plan. In our study, based on the two-dimensional heatmap, we adopted an individualized sub-arc design with 3.25 (range, 2–5) sub-arcs on average, and more reduction of MUs (41.11%) were obtained, as compared with Kim’s work (8).

There are several limitations in this study. Firstly, we have only made a plan comparison, but did not actually perform it on the accelerator, which requires further experiments. Secondly, increasing the number of sub-arc will increase the treatment time and reduce the treatment efficiency. Fortunately, the SACAO method proposed in this study can greatly reduce the MUs, which may alleviate the extension of treatment time to some degree. The measurement of the total treatment time is still needed in future studies. Last but not least, due to the equipment limitations, there is no comparative study with the latest HyperArc technology. In future work, we will try to conduct a comparative study with HyperArc and combine this optimization method with hyper-arc.

## 5 Conclusion

An algorithm of SACAO was developed and applied in single-isocenter coplanar VMAT SRS for multiple metastases. The SACAO method showed certain superiority in improving the CI, HI, and GI of the targets as well as normal tissue sparing. In particular, SACAO has the potential to promote treatment efficiency with respect to field size and MU. The results of the present study suggest that significant advantages could be achieved by using the SACAO algorithm in single-isocenter VMAT SRS of patients with multiple brain metastases.

## Data availability statement

The raw data supporting the conclusions of this article will be made available by the authors, without undue reservation.

## Ethics statement

The studies involving human participants were reviewed and approved by Institutional review board of Zhongnan Hospital of Wuhan University. The patients/participants provided their written informed consent to participate in this study.

## Author contributions

JS: participation in the whole work; generating treatment plans; drafting of the article; data analysis; final approval of the version to be published. ZD: participation in the whole work; perception and design; drafting of the article; data analysis; final approval of the version to be published. JY: drafting and final approval of the version to be published. QY: data analysis; drafting of the article. CC: data analysis; drafting of the article. KK: participation in the whole work; drafting of the figure; data analysis; HL: drafting of the article. CX: drafting of the article. XW: designing the project; drafting and final approval of the version to be published. All authors contributed to the article and approved the submitted version.

## Funding

This study was sponsored by Sanming Project of Medicine in Shenzhen (SZSM201612063), the Shenzhen Key Medical Discipline Construction Fund (SZXK013), the Basic and Applied Basic Research Foundation of Guangdong Province (Grant No. 2020A1515110335), and Shenzhen Postdoctoral Research Funds (Grant No. 25005).

## Conflict of interest

The authors declare that the research was conducted in the absence of any commercial or financial relationships that could be construed as a potential conflict of interest.

## Publisher’s note

All claims expressed in this article are solely those of the authors and do not necessarily represent those of their affiliated organizations, or those of the publisher, the editors and the reviewers. Any product that may be evaluated in this article, or claim that may be made by its manufacturer, is not guaranteed or endorsed by the publisher.
